# Sodium-glucose co-transporter 2 inhibitors in heart failure with mildly reduced or preserved ejection fraction: an updated systematic review and meta-analysis

**DOI:** 10.1186/s40001-022-00945-z

**Published:** 2022-12-29

**Authors:** Yintang Wang, Tong Gao, Chang Meng, Siyuan Li, Lei Bi, Yu Geng, Ping Zhang

**Affiliations:** 1 Department of Cardiology, Beijing Tsinghua Changgung Hospital, School of Clinical Medicine, Tsinghua University, NO. 168 Litang Road, Changping District, Beijing, 102218 People’s Republic of China; 2 Department of Emergency, Emergency General Hospital, Beijing, People’s Republic of China

**Keywords:** Sodium-glucose co-transporter (SGLT2) inhibitors, Heart failure (HF), Heart failure with mildly reduced ejection fraction (HFmrEF), Heart failure with preserved ejection fraction (HFpEF), Outcomes

## Abstract

**Objectives:**

Heart failure with mildly reduced ejection fraction (HFmrEF)  or  heart failure with preserved ejection fraction (HFpEF)  are associated with significant morbidity and mortality, as well as growing health and economic burden. Sodium-glucose co-transporter 2 (SGLT2) inhibitors are very promising for the outcome improvement of patients with HFpEF or HFmrEF. The meta-analysis was performed to investigate the effects of SGLT2 inhibitors in HFpEF or  HFmrEF, by pooling data from all clinically randomized controlled trials (RCTs) available to increase power to testify.

**Methods:**

Studies were searched in electronic databases from inception to November, 2022. We performed a meta-analysis to estimate the effect of SGLT2 inhibitors on clinical endpoints in patients with HFpEF or HFmrEF, using trial-level data with consistent endpoint definitions. The primary outcome was the composite of heart failure (HF) hospitalization or cardiovascular death. Hazard ratio (HR) was pooled with 95% confidence interval (CI) for dichotomous data. This study was registered with INPLASY 2022110095.

**Results:**

Six studies involving 15,989 participants were included into the final analysis. Pooled analyses revealed that SGLT2 inhibitors significantly reduced the composite of HF hospitalization or cardiovascular death [HR: 0.79 (0.72–0.85); *I*^2^ = 0%; *P* < 0.00001] and HF hospitalizations [HR: 0.74 (0.67–0.82); *I*^2^ = 0%; *P* < 0.00001]. This finding was seen in both HFmrEF trials [HR: 0.76 (0.67–0.87); *I*^2^ = 49%; *P* < 0.0001] and HFpEF subgroup studies [HR: 0.70 (0.53–0.93); *I*^2^ = 0%; *P* = 0.01]. The incidence of any serious adverse events [OR: 0.89 (0.83–0.96); *I*^2^ = 0%; *P* = 0.002] was significantly lower in the SGLT2 inhibitor arm. No significant differences were observed between the two groups with regard to cardiovascular death and all-cause death.

**Conclusions:**

This meta-analysis of patients with heart failure of left ventricular ejection fraction (LVEF) > 40% showed that SGLT2 inhibitors significantly reduce the risk of  the composite of cardiovascular death and hospitalization for heart failure, but not cardiovascular death and all-cause death. Nevertheless, given that SGLT2 inhibitors may reduce the risk of hospitalization for heart failure, they should be considered the fundamental treatment for all patients with HFpEF or  HFmrEF.

**Supplementary Information:**

The online version contains supplementary material available at 10.1186/s40001-022-00945-z.

## Introduction

Heart failure (HF) is increasing as the aging population, improved survival after myocardial infarction, and improved treatment and survival of patients with HF [1–3]. Heart failure with preserved ejection fraction (HFpEF), left ventricular ejection fraction (LVEF) ≥ 50%, is highly prevalent, accounting for up to 50% of all patients with HF, while the prevalence of heart failure with mildly reduced ejection fraction (HFmrEF) (LVEF 40%–49%) within the overall population of patients with HF is 10%–25% [4, 5]. Notably, patients with HFpEF bear an extremely debilitating symptoms and physical limitations [6]. HFpEF and HFmrEF are associated with significant morbidity and mortality, as well as growing health and economic burden.

HFpEF is characterized with heterogeneous etiologies and sub-phenotypes, making it difficult for a single drug to be applied universally [5, 7]. Thus, therapeutic options for patients with HFpEF and HFmrEF are limited. According to the current guidelines, diuretics are recommended as needed for the symptomatic patients with HFpEF to relieve the symptoms [8, 9]. To date, the wide range of pharmacotherapies tested have had minimal impact on the improvement of the outcomes in patients with HFpEF or  HFmrEF. The research for a pharmacotherapeutic agent that would improve the “hard endpoints,” such as mortality and major adverse cardiac events, is always a critical unmet need, in order to help the management of these patients [10].

Sodium-glucose co-transporter 2 (SGLT2) inhibitor is an antidiabetic class category that acts by blocking glucose resorption in the proximal tubule of the kidney promoting glucosuria [11]. SGLT2 inhibitors have been shown their cardioprotective and renoprotective effects in various diseases, including type 2 diabetes, chronic kidney disease, and heart failure [12–14]. Although SGLT2 inhibitors are very promising for the outcome improvement of patients with HFpEF or  HFmrEF, as shown in the recently released literatures [15, 16]. However, the recommendations for SGLT2 inhibitors in the updated guidelines are absent or weak in HFpEF and HFmrEF (class II), while they are strongly recommended in heart failure with reduced ejection fraction (class I) [8, 9]. Thus, it is urgent for more evidence-based medicine to provide more certainties.

More recently, some meta-analysis focused on this topic. In a meta-analysis of randomized controlled trials (RCTs) without Dapagliflozin Evaluation to Improve the LIVEs of Patients With PReserved Ejection Fraction Heart Failure (DELIVER), the beneficial effects of SGLT2 inhibitors were found to reduce cardiovascular mortality and hospitalization for heart failure, but not overall mortality in patients with HFpEF [17]. Similarly, Muthiah et al. conducted a meta-analysis of two largest trials [DELIVER and Empagliflozin Outcome Trial in Patients with Chronic Heart Failure with Preserved Ejection Fraction (EMPEROR-Preserved)] and implied that SGLT2 inhibitors significantly reduced the risk of composite cardiovascular death or hospitalization for heart failure, but not all-cause death and cardiovascular death [18]. Thus, it was an instrumental time for an updated and a comprehensive meta-analysis to shed some light on these open issues.

The aim of this prespecified meta-analysis is to investigate the effects of SGLT2 inhibitors in HFpEF and HFmrEF, by pooling data from on all clinical RCTs available to increase power to testify. In this meta-analysis, including six placebo-controlled trials involving HFpEF and HFmrEF, the effects of SGLT2 inhibitors on heart failure hospitalizations, mortality outcomes, adverse events, and in several clinically relevant subgroups were estimated.

## Methods

Preferred Reporting Items for Systematic Reviews and Meta-Analyses (PRISMA) guidelines for performing and reporting our current meta-analysis were followed [19]. Our protocol has been registered beforehand on the International Platform of Registered Systematic Review and Meta-analysis Protocols database (Inplasy protocol: INPLASY 2022110095), and is available in full on inplasy.com (https://inplasy.com/inplasy-2022-11-0095). Ethics approval is not required for this study.

### Search strategy

Three independent researchers (Yintang Wang, Yu Geng, and Chang Meng) conducted extensive electronic searches for relevant articles published on November 15, 2022. The database searched included PubMed, Embase, and the Cochrane database. English retrieval use the medical subject title (MeSH) in combination with the following terms to search: "Sodium-glucose transporter 2 inhibitors," "Heart failure." We manually selected relevant RCTs and screened to identify any relevant studies. The detailed search strategy of the literature is shown in Additional file 1: Table S1.

### Inclusion and exclusion

EndNote (X9 version) software was selected for documents management, and duplicate studies were removed manually. Two investigators (Chang Meng and Yu Geng) independently evaluated the eligibility of the identified items. If there were any discrepancies in the inclusion decisions, comprehensive discussion with another author (Yintang Wang) would be conduct until reaching a consensus. The title and summary were filtered for the first time, and qualified articles were reserved for full-text review. Inclusion criteria for studies includes the following: (1) comparison between SGLT2 inhibitors and placebo; (2) included HF patients involving HFpEF or HFmrEF; (3) either RCTs or post hoc analyses of RCTs; and (4) at least one of the predefined outcomes of interest was reported. We excluded studies that did not provide full text, observational studies, and studies were not written in English.

### Outcomes of interest

The primary outcome of interest was the composite of HF hospitalization or cardiovascular death. The other outcomes of interest included all-cause death, cardiovascular death, and HF hospitalization. For the composite of HF hospitalization or cardiovascular death, comparisons between SGLT2 inhibitors and placebo were conducted in HFpEF and HFmrEF, respectively. The serious adverse events and serious adverse events leading to study drug discontinuation were also analyzed.

### Bias and quality assessment

The two researchers independently evaluated, preliminarily selected, and checked the literature data according to the unified and standardized methods, and included them in the literature in strict accordance with the inclusion and exclusion criteria, and then collected information. The quality of selected articles was evaluated according to the quality evaluation standard of Cochrane Reviewer Handbook 5.1.0 [20] (random sequence generation, allocation concealment, blinding of participants and personnel, blinding of outcome assessment, incomplete outcome data, selective reporting, and other bias).

### Data synthesis and analysis

The Review Manager (RevMan, Version 5.3) software was used for meta-analysis. All effect sizes were extracted as point estimates with 95% confidence intervals (CIs). For the time-to-first event endpoints, the meta-analysis included data from Cox proportional hazards models reported as hazard ratios (HRs) and 95% CIs. Data that meet homogeneity (*P* > 0.10 and *I*^2^ ≤ 50%) through heterogeneity test was meta-analyzed with fixed effect model. If homogeneity (*P* ≤ 0.10 or *I*^2^ > 50%) was not met, and heterogeneity cannot be ruled out, random effect model can be used to combine effects, but it should be noted that sensitivity analysis and subgroup analysis were also considered.

## Results

### Search results and study characteristics

**Fig. 1:**
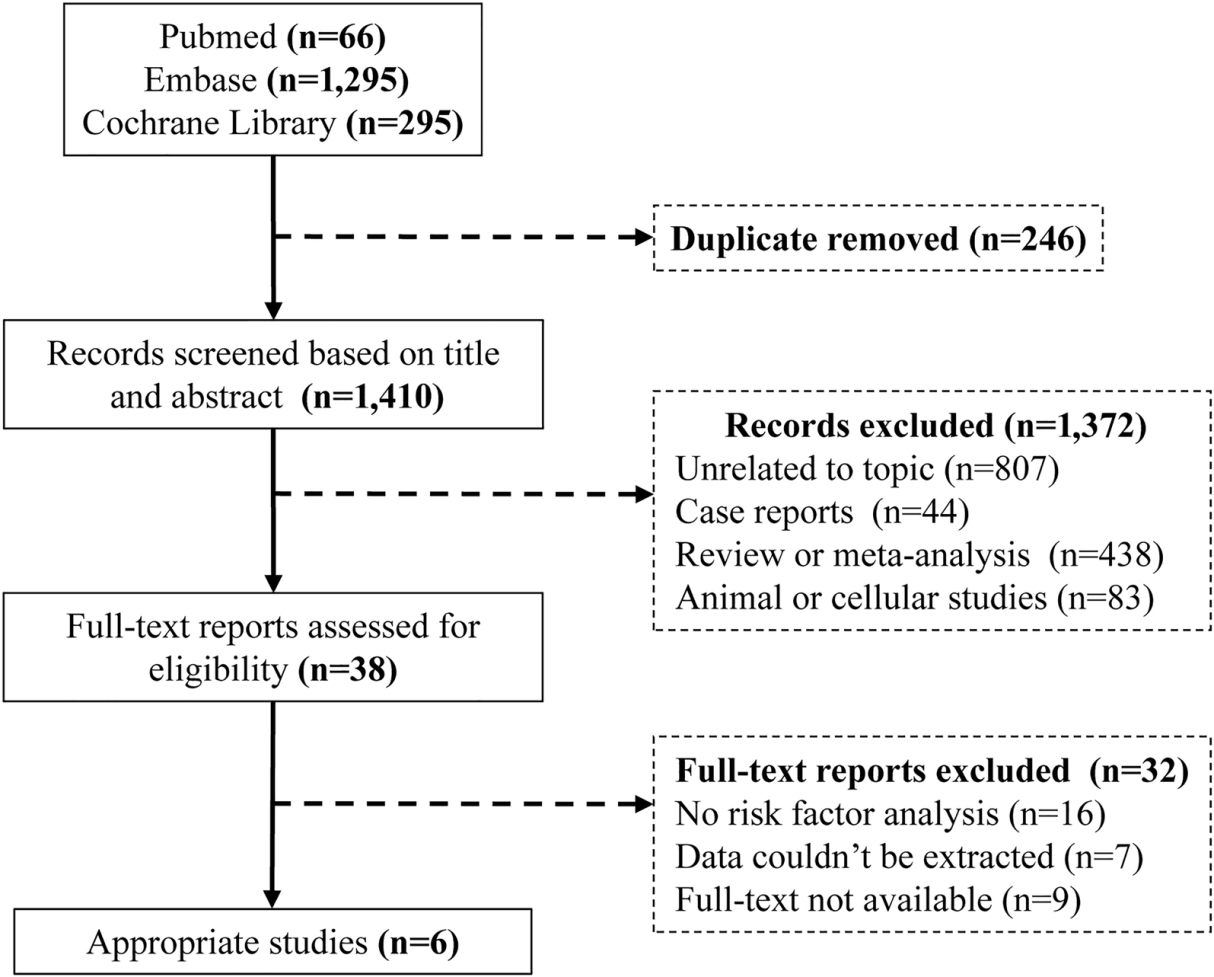
Flow diagram of the study selection process

The flow chart (Fig. 1) summarizes the search and study selection process. A total of 1,656 studies were identified through the electronic searches, of which 246 were excluded due to duplication. 1,372 studies were also excluded after reading the titles and abstracts. The remaining 38 studies were assessed by reading the full texts. Data from 6 RCTs evaluating SGLT2 inhibitors were included.

The overall study population in this meta-analysis included 15,989 HF patients (*N* = 8,177 in the SGLT2 inhibitor arms; *N* = 7,812 in the placebo arms). The characteristics of the included studies are summarized in Table 1. The Sotagliflozin in Patients with Diabetes and Recent Worsening Heart Failure (SOLOIST-WHF) [21], The Dapagliflozin Effect on Cardiovascular Events–Thrombolysis in Myocardial Infarction 58 (DECLARE–TIMI 58) [22], Evaluation of Ertugliflozin Efficacy and Safety Cardiovascular Outcomes Trial (VERTIS-CV) [23], and Sotagliflozin on Cardiovascular and Renal Events in Patients with Type 2 Diabetes and Moderate Renal Impairment Who Are at Cardiovascular Risk (SCORED) [24] trials included only diabetes mellitus (DM) patients. The EMPEROR-Preserved [15] and DELIVER [16] trials included both DM and non-DM patients. The SOLOIST-WHF, EMPEROR-Preserved, and DELIVER trials included both HFmrEF patients and HFpEF patients. In VERTIS-CV and DECLARE-TIMI 58, an ejection fraction > 45% with known HF was considered as HFpEF, while in SOLOIST-WHF, an ejection fraction > 50% was considered HFpEF. All included trials were at low risk of bias (Additional file 1: Fig. S1).

**Table 1:** Design and outcomes of the studies included in the meta-analysis

Num	Research	Total patients	Median follow-up	SGLT2 inhibitor	EF at baseline	DM status At baseline	Primary outcomes
1	EMPEROR-Preserved	5988	26.2 months	Empagliflozin	EF > 40%	DM/non-DM	Cardiovascular death or hospitalization for heart failure
2	SOLOIST-WHF	256	9.2 months	Sotagliflozin	EF ≥ 50%	DM	Death from cardiovascular causes or hospitalization for HF
3	DELIVER	6263	28.1 months	Dapagliflozin	EF > 40%	DM/non-DM	An unplanned hospitalization for HF or an urgent visit for HF, or cardiovascular death
4	DECLARE-TIMI 58	808	50.4 months	Dapagliflozin	EF > 45%	DM	Cardiovascular death or HF hospitalization
5	VERTIS-CV	1007	36.0 months	Ertugliflozin	EF > 45%	DM	The time to first major adverse cardiovascular event
6	SCORED	1667	16 months	Sotagliflozin	EF ≥ 50%	DM	The first occurrence of a major adverse cardiovascular event

EF = ejection fraction, DM = diabetes mellitus, HF = heart failure, SGLT2 Inhibitor = Sodium-glucose co-transporter 2 inhibitors

### Results for the heart failure cohort

**Fig. 2:**
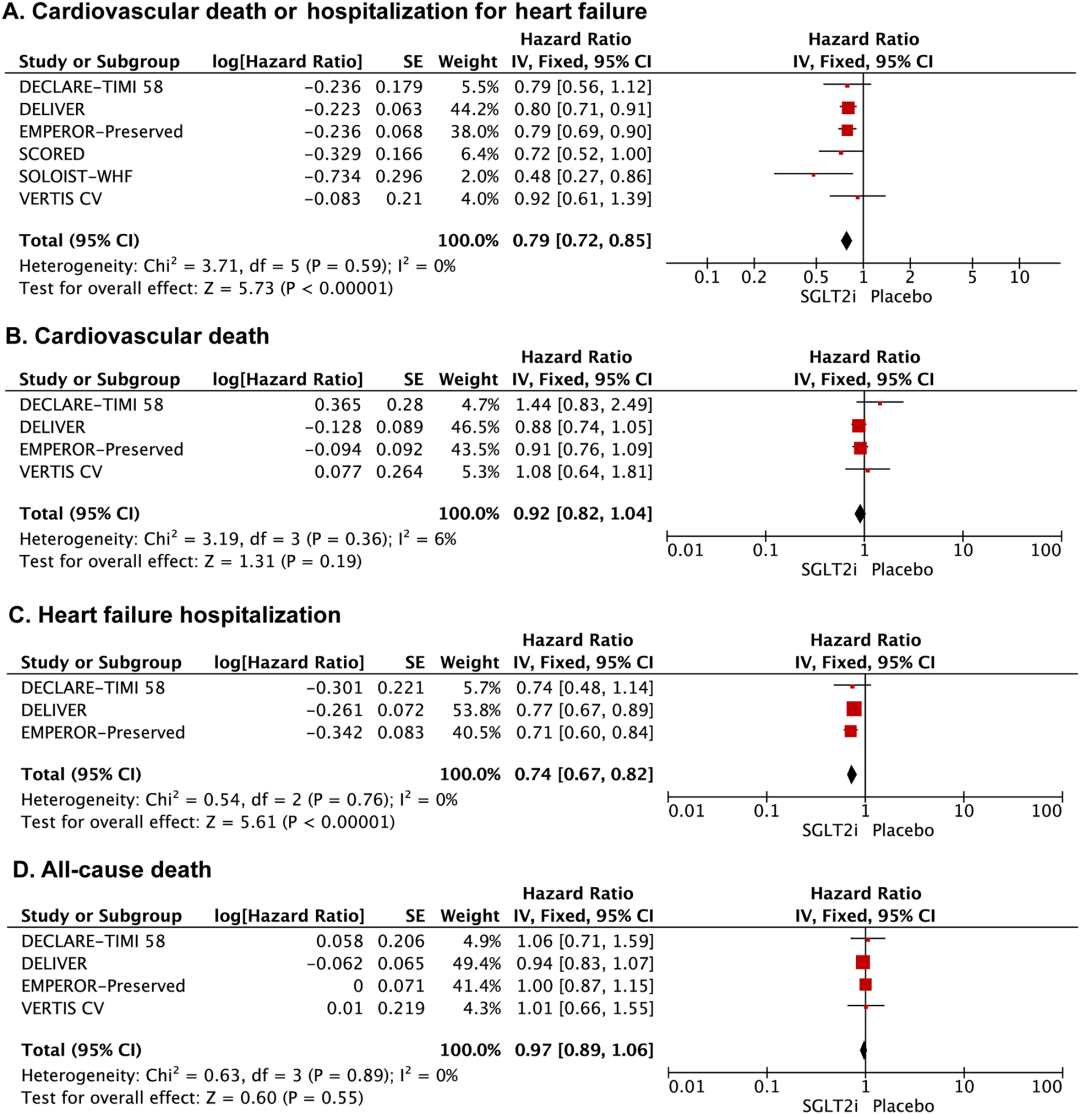
Forest plot for the effect of SGLT2 inhibitors vs placebo on the risk of outcomes for patients with HFmrEF/HFpEF. Forest plot reporting the hazard ratios of the SGLT2 inhibitors vs placebo in patients with HFmrEF/HFpEF: **A** cardiovascular death or hospitalization for heart failure; **B** Cardiovascular death; **C** heart failure hospitalization; **D** All-cause death

**Fig. 3:**
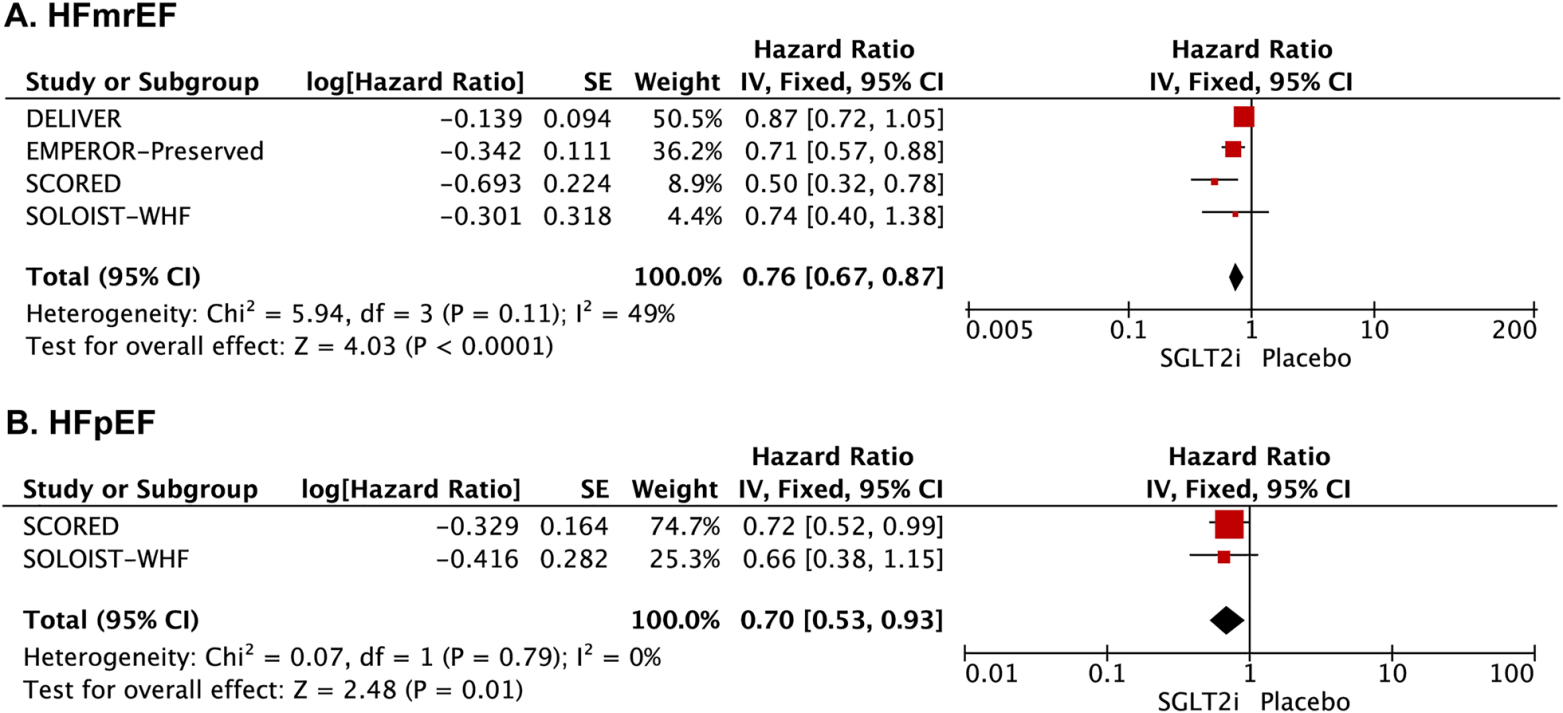
The effect of SGLT2 inhibitors vs placebo on the risk of outcomes for the patients with HFmrEF or HFpEF. Forest plot for the SGLT2 inhibitors versus placebo in patients with heart failure: **A** HFmrEF; **B** HFpEF. HFmrEF = heart failure with preserved ejection fraction; HFpEF = heart failure with mildly reduced ejection fraction

SGLT2 inhibitors significantly reduced the composite of HF hospitalization or cardiovascular death [HR: 0.79 (0.72–0.85); *I*^2^ = 0%; *P* < 0.00001] (Fig. 2A). This finding was seen in both HFmrEF trials [HR: 0.76 (0.67–0.87); *I*^2^ = 49%; *P* < 0.0001] and HFpEF subgroup studies [HR: 0.70 (0.53–0.93); *I*^2^ = 0%; *P* = 0.01] (Fig. 3). SGLT2 inhibitors were associated with a significant reduction in total HF hospitalizations [HR: 0.74 (0.67–0.82); *I*^2^ = 0%; *P* < 0.00001] (Fig. 2C). However, there was no significant difference between the SGLT2 inhibitors and placebo groups for the risk of cardiovascular death [HR: 0.92 (0.82–1.04); *I*^2^ = 6%; *P* = 0.19] (Fig. 2B) and all-case death [HR: 0.97 (0.89–1.06); *I*^2^ = 0%; *P* = 0.55] (Fig. 2D) among HFpEF/HFmrEF patients.

### Safety

**Fig. 4:**
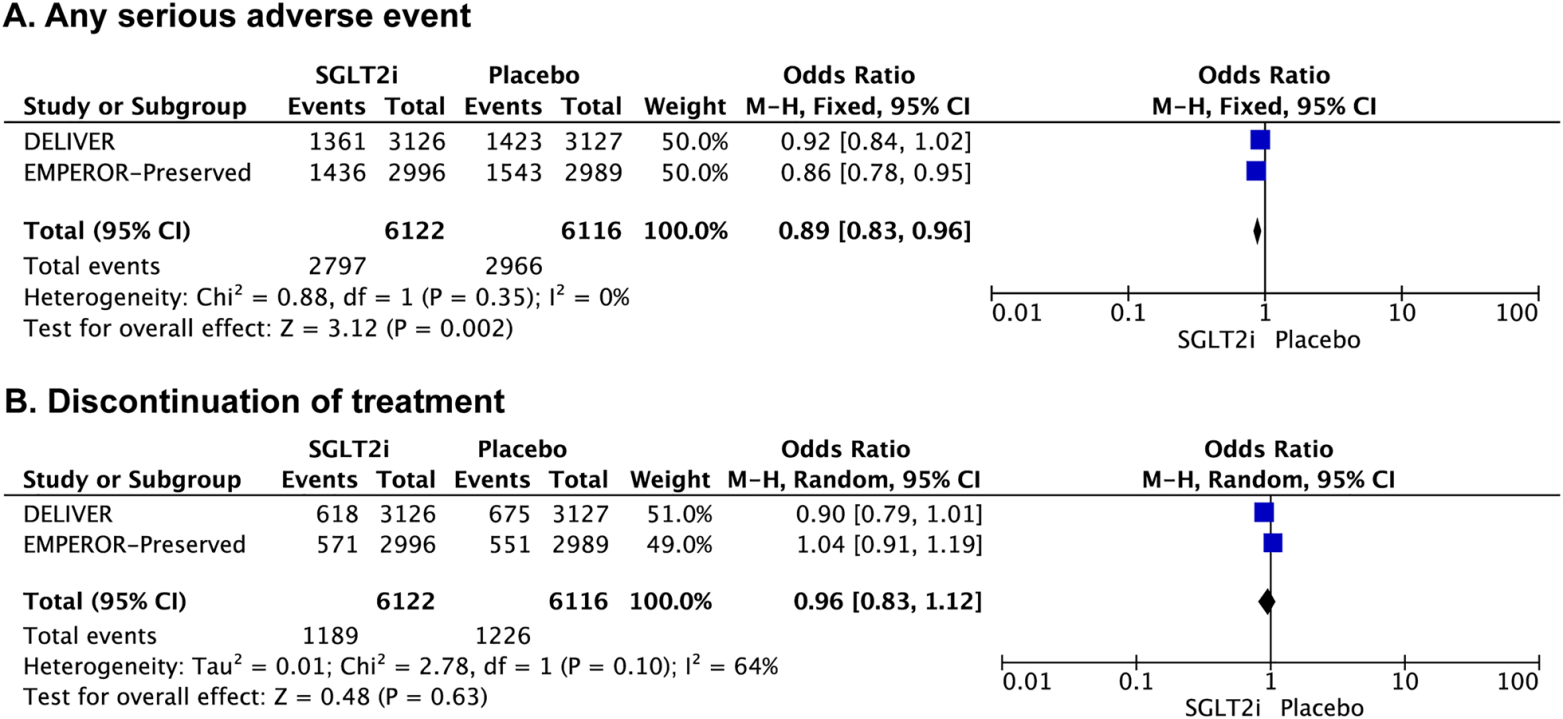
The effect of SGLT2 inhibitors versus placebo on the risk of adverse events for the patients with HFmrEF/HFpEF. Forest plot for the SGLT2 inhibitors vs placebo in patients with HFmrEF/HFpEF: **A** Any serious adverse event; **B** Discontinuation of treatment

In the overall cohort, the incidence of any serious adverse events [OR: 0.89 (0.83–0.96); *I*^2^ = 0%; *P* = 0.002] was significantly lower in the SGLT2 inhibitor arm. There was no significant difference between the SGLT2 inhibitor and placebo groups in the incidence of discontinuation due to adverse events [OR: 0.96 (0.83–1.12); *I*^2^ = 64%; *P* = 0.63] (Fig. 4).

### Sensitivity analysis

In sensitivity analysis, only slight change of risk estimates was observed when removing a study for all outcomes. These results indicated the robustness of the present findings, and that no single study drove the summary effects.

## Discussion

As we all know, HFpEF is the main type of heart failure, and its prevalence is increasing year by year [4, 5]. Although patients with HFpEF have a normal LVEF, the prognosis is poor due to the impaired myocardial contractility and left ventricular systolic function [25]. Over the past few years, multiple clinical trials of the cardiovascular benefits of SGLT2 inhibitors have had mixed results. This meta-analysis, including six large trials of four kinds of SGLT2 inhibitors in HFpEF and HFmrEF, showed that the SGLT2 inhibitors robustly reduced the composite of cardiovascular death or hospitalization for heart failure, supporting their beneficial effects as a foundational therapy for patients with HFpEF or HFmrEF. The clinical benefit of SGLT2 inhibitors appeared consistent across a broad range of patients, regardless of the level of LVEF. However, the risk of neither all-cause death nor cardiovascular death were decreased in HFpEF and HFmrEF, suggesting that other complications related to heart failure might play an important role.

Although there have been similar meta-analyses on this issue before, they all have certain limitations. Compared with the meta-analysis conducted by Vaduganathan et al. [18], we focused on patients with heart failure of LVEF > 40%. Despite reaching the similar conclusion, we provided dedicated evidence and more power with a largest sample size so far to support the use of SGLT2 inhibitors in patients with HFpEF and HFmrEF. Another meta-analysis conducted by *Tsampasian *et al. also reached similar conclusions, unfortunately lacking data of recently published DELIVER trial [17]. This meta-analysis was updated based on previous studies to include the newly published study, DELIVER, which included 6,263 patients with LVEF > 40%. It can provide more data support for this meta-analysis. Compared with the meta-analysis published by *Younes *et al., which only focused on the safety outcomes [26], our present meta-analysis increased the analysis of clinical primary effective endpoints, which is of great significance and provides evidence for clinicians to make decisions in real-world practice.

SGLT2 inhibitors decreased the risk of cardiovascular death or hospitalization for heart failure in patients with HFpEF or HFmrEF. However, they did not reduce the risk of isolated cardiovascular death or isolated all-cause death. This means that the beneficial effects of SGLT2 inhibitors mainly derived from the reduction in the incidence of hospitalization for heart failure in patients with HFpEF or HFmrEF. The exact mechanism of this effect is undetermined. However, multiple theories have been proposed. Hypertension, hyperglycemia and obesity are typically complicated with HFpEF patients and are related to the increased risk of morbidity and mortality [27]. SGLT2 inhibitors are beneficial in the control of blood pressure, blood sugar, and body mass index, so they could bring benefits to patients with HFpEF [28, 29]. SGLT2 inhibitors also have a diuretic effect with a predilection to reduce interstitial volume [30]. Endothelial dysfunction mediated by inflammatory response and oxidative stress may also damage cardiomyocyte function in patients with HFpEF [31]. There is growing evidence that SGLT2 inhibitors can reduce inflammation and oxidative stress, thereby reducing the incidence of adverse cardiovascular events [32, 33]. In addition, SGLT2 inhibitors can relieve the symptoms of HFpEF by interfering with metabolic pathways. SGLT2 inhibitors can induce ketogenic metabolism, thus using energy-efficient ketone bodies to increase myocardial energy source and improve myocardial function [34]. Additionally, SGLT2 inhibitors may have an antifibrotic effect on the myocardium and cause natriuresis [35]. Therefore, patients with HFpEF may benefit from SGLT2 inhibitors through a variety of mechanisms.

Although, data acquisition and precise definition of these adverse events varied between the different trials included. In this meta-analysis, we summarized the safety endpoint of SGLT2 inhibitors in patients with HFpEF and HFmrEF. SGLT2 inhibitors were safe and well tolerated, without excess in serious adverse events. SGLT2 inhibitors were associated with a significant decrease in severe adverse events. This is consistent with the results of the two included trials and the previous meta-analysis [17, 18, 26]. Severe adverse events remained significantly lower after running the sensitivity analysis.

We recognize that our study also has limitations. Firstly, there were differences in baseline characteristics, including age, LVEF, and length of follow-up, which could lead to heterogeneity. We used a random effects model and performed a sensitivity analysis to adjust for this limitation. Secondly, not all trials involving patients with HFpEF and HFmrEF reported cardiovascular death, heart failure hospitalization, and all-cause death. Fortunately, the data from DELIVER and EMPEROR-Preserved which were two large studies focused on HFpEF and HFmrEF populations were available. Thirdly, sotagliflozin inhibits SGLT1 and SGLT2, while dapagliflozin and empagliflozin only inhibits SGLT2. Finally, we did not observe “positive results” for “hard endpoints,” such as all-cause death and cardiovascular death. However, by focusing exclusively on clinical trials in patients with HFpEF, regardless of their diabetes status, this meta-analysis was able to demonstrate cardiovascular benefits of SGLT2i in patients with HFpEF and HFmrEF. In order to draw robust conclusions regarding these efficacy outcomes, further larger trials are needed to evaluate the effect of the SGLT2 inhibitors in a sufficient number of patients with HFpEF or HFmrEF.

## Conclusions

In conclusion, this meta-analysis of patients with heart failure of LVEF>40% showed that SGLT2 inhibitors significantly reduced the risk of the composite of cardiovascular death and hospitalization for heart failure, but not cardiovascular death and all-cause death. Nevertheless, given that SGLT2 inhibitors may reduce the risk of hospitalization for heart failure, they should be considered the fundamental treatment for all patients with HFpEF or HFmrEF.

## Supplementary Information

**Additional file 1**: Table S1 and Figure S1.

## Data Availability

The data sets used or analyzed during the current study are available from the corresponding author on request.

## Abbreviations

HF: Heart failure
HFmrEF: Heart failure with mildly reduced ejection fraction
HFpEF: Heart failure with preserved ejection fraction
HRs: Hazard ratios
LVEF: Left ventricular ejection fraction
OR: Odds ratio
PRISMA: Preferred Reporting Items for Systematic Reviews and Meta-Analyses
Cis: Confidence intervals
RCTs: Randomized controlled trials
SGLT2: Sodium-glucose co-transporter 2

## Code availability

This article is distributed under the terms of the Creative Commons Attribution License, which permits unrestricted use and redistribution provided that the original author and source are credited.
