# Supplementary material for: Sodium-glucose co-transporter 2 inhibitors in heart failure with mildly reduced or preserved ejection fraction: an updated systematic review and meta-analysis
Source: Eur J Med Res. 2022 Dec 29;27:314. doi: 10.1186/s40001-022-00945-z (PMC9798580; doi:10.1186/s40001-022-00945-z)

**Table.S1 Search strategy**

| Electronic database | Search strategy |
| --- | --- |
| PubMed (NCBI) | ((Search: ((((((((((((Sodium-Glucose Transporter 2 Inhibitors[MeSH Terms]) OR (Sodium Glucose Transporter 2 Inhibitors[Title/Abstract])) OR (SGLT-2 Inhibitors[Title/Abstract])) OR (SGLT 2 Inhibitors[Title/Abstract])) OR (SGLT2 Inhibitors[Title/Abstract])) OR (Sodium-Glucose Transporter 2 Inhibitor[Title/Abstract])) OR (Sodium Glucose Transporter 2 Inhibitor[Title/Abstract])) OR (SGLT2 Inhibitor[Title/Abstract])) OR (Inhibitor, SGLT2[Title/Abstract])) OR (Gliflozin[Title/Abstract])) OR (SGLT-2 Inhibitor[Title/Abstract])) OR (Inhibitor, SGLT-2[Title/Abstract])) OR (SGLT 2 Inhibitor[Title/Abstract])) AND (((((((((((((((heart failure[MeSH Terms]) OR (Cardiac Failure[Title/Abstract])) OR (Myocardial Failure[Title/Abstract])) OR (Heart Failure, Left-Sided[Title/Abstract])) OR (Heart Failure, Left Sided[Title/Abstract])) OR (Left-Sided Heart Failure[Title/Abstract])) OR (Left Sided Heart Failure[Title/Abstract])) OR (Heart Failure, Right-Sided[Title/Abstract])) OR (Heart Failure, Right Sided[Title/Abstract])) OR (Right-Sided Heart Failure[Title/Abstract])) OR (Right Sided Heart Failure[Title/Abstract])) OR (Congestive Heart Failure[Title/Abstract])) OR (Heart Failure, Congestive[Title/Abstract])) OR (Heart Decompensation[Title/Abstract])) OR (Decompensation, Heart[Title/Abstract]))) AND (randomized controlled trial[Publication Type] OR randomized[Title/Abstract] OR placebo[Title/Abstract]) |
| Embase | #1 'heart failure'/exp  #2 'cardiac failure':ab,ti OR 'myocardial failure':ab,ti OR 'heart failure, left-sided':ab,ti OR 'heart failure, left sided':ab,ti OR 'left-sided heart failure':ab,ti OR 'left sided heart failure':ab,ti OR 'heart failure, right-sided':ab,ti OR 'heart failure, right sided':ab,ti OR 'right-sided heart failure':ab,ti OR 'right sided heart failure':ab,ti OR 'congestive heart failure':ab,ti OR 'heart failure, congestive':ab,ti OR 'heart decompensation':ab,ti OR 'decompensation, heart':ab,ti  #3 #1 OR #2  #4 'sodium glucose cotransporter 2 inhibitor'/exp  #5 'sodium glucose transporter 2 inhibitors':ab,ti OR 'sglt-2 inhibitors':ab,ti OR 'sglt 2 inhibitors':ab,ti OR 'sglt2 inhibitors':ab,ti OR 'sodium-glucose transporter 2 inhibitor':ab,ti OR 'sodium glucose transporter 2 inhibitor':ab,ti OR 'sglt2 inhibitor':ab,ti OR 'inhibitor, sglt2':ab,ti OR 'gliflozins':ab,ti OR 'gliflozin':ab,ti OR 'sglt-2 inhibitor':ab,ti OR 'inhibitor, sglt-2':ab,ti OR 'sglt 2 inhibitor':ab,ti  #6 #4 OR #5  #7 'randomized controlled trial':ab,ti OR 'randomized':ab,ti OR 'placebo':ab,ti  #8 #3 AND #6 AND #7 |
| Cochrane | #1 MeSH descriptor: [Sodium-Glucose Transporter 2 Inhibitors] explode all trees  #2 (Sodium Glucose Transporter 2 Inhibitors):ti,ab,kw OR (SGLT-2 Inhibitors):ti,ab,kw OR (SGLT 2 Inhibitors):ti,ab,kw OR (SGLT2 Inhibitors):ti,ab,kw OR (Sodium-Glucose Transporter 2 Inhibitor):ti,ab,kw OR (Sodium Glucose Transporter 2 Inhibitor):ti,ab,kw OR (SGLT2 Inhibitor):ti,ab,kw OR (Inhibitor, SGLT2):ti,ab,kw OR (Gliflozins):ti,ab,kw OR (Gliflozin):ti,ab,kw OR (SGLT-2 Inhibitor):ti,ab,kw OR (Inhibitor, SGLT-2):ti,ab,kw OR (SGLT 2 Inhibitor):ti,ab,kw  #3 MeSH descriptor: [Heart Failure] explode all trees  #4 (Infarction, Myocardial):ti,ab,kw OR (Infarctions, Myocardial):ti,ab,kw OR (Myocardial Infarctions):ti,ab,kw OR (Cardiovascular Stroke):ti,ab,kw OR (Cardiovascular Strokes):ti,ab,kw OR (Stroke, Cardiovascular):ti,ab,kw OR (Strokes, Cardiovascular):ti,ab,kw OR (Myocardial Infarct):ti,ab,kw OR (Infarct, Myocardial):ti,ab,kw OR (Infarcts, Myocardial):ti,ab,kw OR (Myocardial Infarcts):ti,ab,kw OR (Heart Attack):ti,ab,kw OR (Heart Attacks):ti,ab,kw  #5 #1 OR #2  #6 #3 OR #4  #7 #5 AND #6 |

**Supplement Figure 1.** **Risk of bias graph**


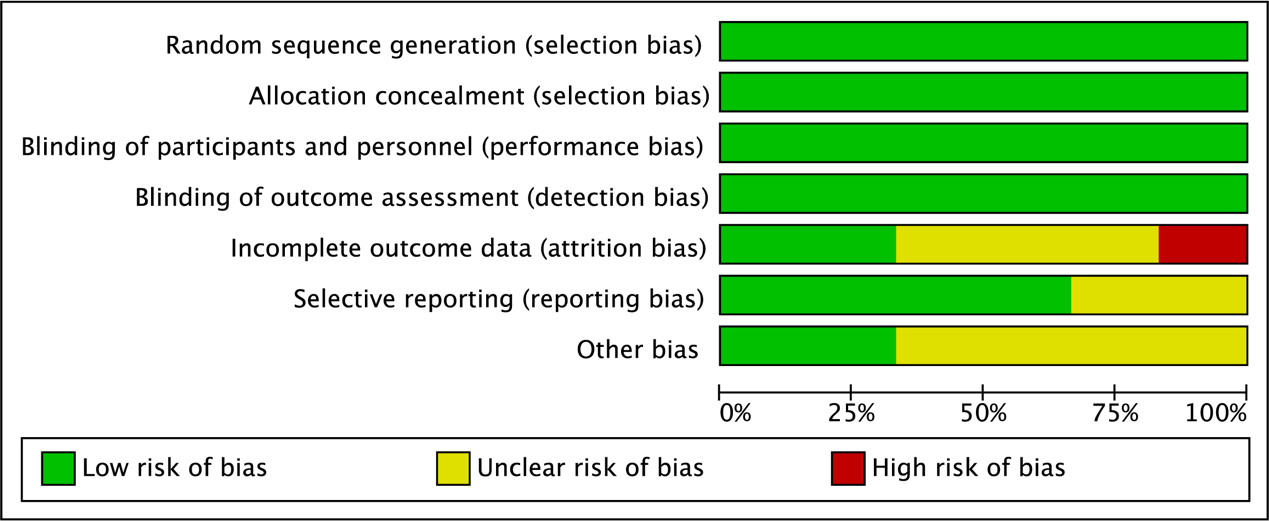

Supplement: Supplementary file 1 — Additional file 1: Table S1 and Figure S1. [file 40001_2022_945_MOESM1_ESM.docx]
